# Convolutional neural network model based on radiological images to support COVID-19 diagnosis: Evaluating database biases

**DOI:** 10.1371/journal.pone.0247839

**Published:** 2021-03-01

**Authors:** Caio B. S. Maior, João M. M. Santana, Isis D. Lins, Márcio J. C. Moura

**Affiliations:** 1 CEERMA - Center for Risk Analysis, Reliability Engineering and Environmental Modeling, Universidade Federal de Pernambuco, Recife, Brazil; 2 Department of Production Engineering, Universidade Federal de Pernambuco, Recife, Brazil; Taipei Medical University, TAIWAN

## Abstract

As SARS-CoV-2 has spread quickly throughout the world, the scientific community has spent major efforts on better understanding the characteristics of the virus and possible means to prevent, diagnose, and treat COVID-19. A valid approach presented in the literature is to develop an image-based method to support COVID-19 diagnosis using convolutional neural networks (CNN). Because the availability of radiological data is rather limited due to the novelty of COVID-19, several methodologies consider reduced datasets, which may be inadequate, biasing the model. Here, we performed an analysis combining six different databases using chest X-ray images from open datasets to distinguish images of infected patients while differentiating COVID-19 and pneumonia from ‘no-findings’ images. In addition, the performance of models created from fewer databases, which may imperceptibly overestimate their results, is discussed. Two CNN-based architectures were created to process images of different sizes (512 × 512, 768 × 768, 1024 × 1024, and 1536 × 1536). Our best model achieved a balanced accuracy (BA) of 87.7% in predicting one of the three classes (‘no-findings’, ‘COVID-19’, and ‘pneumonia’) and a specific balanced precision of 97.0% for ‘COVID-19’ class. We also provided binary classification with a precision of 91.0% for detection of sick patients (i.e., with COVID-19 or pneumonia) and 98.4% for COVID-19 detection (i.e., differentiating from ‘no-findings’ or ‘pneumonia’). Indeed, despite we achieved an unrealistic 97.2% BA performance for one specific case, the proposed methodology of using multiple databases achieved better and less inflated results than from models with specific image datasets for training. Thus, this framework is promising for a low-cost, fast, and noninvasive means to support the diagnosis of COVID-19.

## 1 Introduction

The 2019-20 coronavirus pandemic is a public health emergency of global concern. Indeed, the coronavirus disease 2019 (COVID-19) is highly contagious and may lead to acute respiratory distress, severe pneumonia, multiple organ failure and death. Its symptoms include fever, dry cough, and shortness of breath; until now, there has been no specific treatment recommended for COVID-19. General recommendations to avoid the spread of this coronavirus include frequent hand-washing, mask-wearing, social distancing, and avoiding close contact with infected people [1].

COVID-19 is commonly diagnosed with polymerase chain reaction (PCR) and serological testing [2], but there is a shortage of required material and specialized personnel to perform these tests mainly in late-stricken regions (e.g., Latin America and Africa). Moreover, PCR can have relatively poor sensitivity [3–5]. Hence, alternative methods to support the diagnosis of COVID-19 are urgently needed.

Recent studies have suggested that COVID-19 could be detected using noninvasive radiological images [3, 4]. For example, computed tomography (CT) images can be used to detect certain manifestations in the lung associated with COVID-19 [6, 7]. Huang *et al*. [8] found that chest CT was indispensable in selecting candidates for quarantine in Wuhan and other places in China. Guan *et al*. [9] mentioned that the majority of COVID-19-positive patients presented abnormal CT results such as ground-glass opacity and bilateral abnormalities. Li and Xia [10] found that chest CT had a low rate of missed diagnosis of COVID-19 being useful as a standard method for the rapid diagnosis and improving the management of patients. Despite the arduous differentiation caused by certain similar imaging features between COVID-19 and other types of respiratory syndromes, CT analysis could indeed serve as an effective way to screen and diagnose COVID-19 [11].

Generally, CT is a more precise technique for imaging the chest and has a higher sensitivity and efficiency than chest X-rays (CXR) [8]. However, the use of CT for COVID-19 detection places a considerable burden on radiology units and is an immense challenge for infection control in the CT suite; thus, portable CXR devices can help minimize the risk of cross-infection [12]. In fact, X-ray images are part of routine patient care and are still the primary imaging test for pneumonia due to the shorter time and lower cost and radiation exposure versus CT [13]. Moreover, Wong *et al*. [12] demonstrated that the common CT findings in COVID-19 patients can also be appreciated on CXR.

Recently, artificial intelligence (AI) models have provided successful results in analyzing data in a medical context. In fact, new machines have already been manufactured with AI models that perform similarly to experts in specific evaluation tasks [14, 15]. Moreover, a relevant application is the adoption of AI systems to extract information from medical imaging with the ultimate purpose of creating tools to reduce diagnostic errors, enhance efficiency, and reduce costs. These systems are typically embedded in image-based decision-support systems to provide aid for imaging professionals [16]. Medical imaging enables specific capabilities, including risk assessment, detection, diagnosis, prognosis, therapy response, and multi-omics disease discovery [17]. Their inherent automatic processing requires fewer laboratory infrastructure and supplies, as well as less health personnel. Hence, medical image analysis could also be beneficial in the diagnosis of COVID-19 [18].

In this context, AI-based models that use radiological medical images as input are an alternative to automate the detection of COVID-19 in patients for clinical analysis and diagnostic support. Exams based on radiological images are consolidated and can ease the burdens of highly specialized equipment for data generation (e.g., CXR image). Moreover, because CXR is a standard exam, it is possible to develop and test models based on previously available data. This can avoid the distress of disturbing patients to produce new information.

Even though previous works have developed AI-based models to identify COVID-19 in patients with CT and/or CXR images, most of them have limitations such as (i) use of private data preventing reproducibility; (ii) creation based on a few databases, which may not be diverse enough to truly detect features from COVID-19 and other illnesses; and (iii) not being flexible enough to completely exploit images of superior quality, e.g., of higher dimensions if they become available. Therefore, we offer here a broader discussion about the importance of adequately using various open datasets to feed the AI model and the application of data augmentation (DA) for classes with a small amount of data (images related to COVID-19, in this work). Thus, we expect to avoid biasing the model towards detecting datasets due to the characteristic features of each data source as well as fully exploiting available images from COVID-19 cases, which are still scarce.

Specifically, in this paper, we developed convolutional neural network (CNN)-based models to distinguish healthy and infected patients differentiating the latter for COVID-19 and pneumonia infections via radiological images (i.e., CXR) available from six different open databases. They enabled screening features compatible with COVID-19 infections and expanded the forms of detection of COVID-19 to support more accurate diagnosis. After training, the multiclassification models should correctly differentiate patients in new and unseen images within seconds or less. The speed with which they provide a result is very significant given that mass testing is critical but currently slow. Also, the proposed CNN-based models can be part of a computer-aided detection (CADe) and a computer-aided diagnosis (CADx) framework [17]. Indeed, our models do not depend on previously trained models and therefore are not constrained to predefined image dimensions before training.

The remainder of this article is organized as follows. In Section 2, we provide a brief overview of CNN and a literature review of current diagnostic models for COVID-19 based on CT and, especially, CXR images. Section 2 also introduces a discussion about the use of diverse datasets to enhance model robustness and performance. Section 3 details the considered databases of CXR images as well as the proposed methodology. We present and discuss the obtained results using the proposed models comparing the effect of different models, dimensions, and datasets in Section 4. Finally, Section 5 summarizes the main findings of the work and provides some concluding remarks.

## 2 Overview of CNN and COVID-19 diagnostic models

The field of computational learning includes machine learning (ML) and deep learning (DL), aiming to detect meaningful patterns in data automatically and to solve problems, which are impossible (or impractical) to be represented by explicit algorithms [19]. Traditional ML techniques have already been successfully applied to a diversity of pattern recognition and regression tasks [20–22]. DL learns high-level abstractions in data by utilizing hierarchical architectures [23]. It combines several layers of nodes to build up progressively more abstract representations of the data making it possible to learn concepts such as object categories directly from raw sensory data [24]. The current success of DL is directly related to the spread of cheap, multi-processor graphics cards, or Graphics Processing Units (GPUs) that increase the speed and decrease the training time for creating a DL model [25].

**Fig 1:**
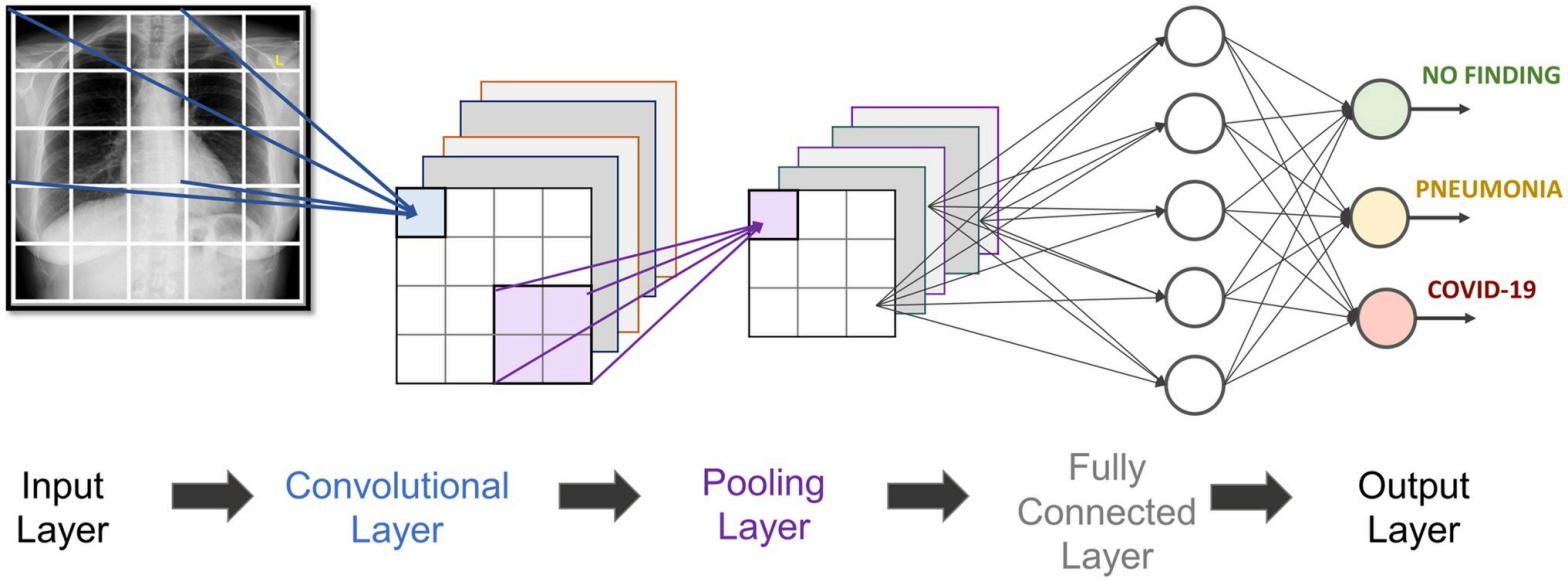
Generic two-dimensional CNN for COVID-19 detection.

CNN is used in pattern recognition with superior feature learning capabilities, being a suitable model to deal with image data [26]. Indeed, CNN is a dominant architecture of DL for image classification and can rival human accuracies in many tasks [27]. CNN uses hierarchical layers of tiled convolutional filters to mimic the effects of human receptive fields on feedforward processing in the early visual cortex thereby exploiting the local spatial correlations present in images while developing robustness to natural transformations such as changes of viewpoint or scale [24]. A CNN-based model generally requires a large set of training samples to achieve good generalization capabilities. Its basic structure is represented as a sequence of Convolutional—Pooling—Fully Connected Layers (Fig 1) possibly with other intermediary layers for normalization and/or dropout.

In fact, CNN has already been successfully applied to medical tasks such as the diagnosis of retinopathy [28], pneumonia [29], cardiomegaly [30] as well as several types of cancer [31]. Due to its ability to extract information from visual features, CNN can be applied to the task of detecting COVID-19 in patients, based on chest CT and/or X-ray images. However, the literature for COVID-19 detection based on CNN is limited, with many of them still provided in non-peer review repositories (e.g., arXiv, medRxiv).

Considering CT images as an input, Li *et al*. [11] presented COVNET, a tuned model of the well-known CNN-based ResNet50 [32], with promising results for COVID-19 diagnosis based on data gathered from six hospitals. The authors originally mentioned the availability of the database in the paper, and a later update reported that the authors did not get permission to share the data and model. Based on a pre-treained ResNet18 model and an attention mechanism, Xu *et al*. [33] used 618 CT images from three distinct hospitals to train an model and classify images as ‘healthy’, influenza-A viral pneumonia and COVID-19 cases. However, despite the good description of data, the dataset is not provided. Singh *et al*. [34] used a multi-objective differential evolution (MODE) to set initial parameters of a CNN to classify the COVID-19-suspected patients as infected or not arguing that CNN provides good results, but it suffers from hyperparameter tuning issues. However, the authors did not explicitly state which database was used; hence, it is not possible to compare their results. Wang *et al*. [35] collected 1065 CT images of COVID-19 and viral pneumonia patients and adopted CNN to demonstrate the proof-of-principle for extracting radiological features, but the images are not available. Butt *et al*. [36] technically reviewed multiple CNN models to classify CT samples with COVID-19, influenza, viral pneumonia, or no infection based on a database. Once again, the images are not available.

The literature is also limited for CXR images (main works are presented in Table 1). Sedik *et al*. (2020) [37] performed binary classification (‘no-finding’ vs COVID-19), with an interesting analysis of DA methods. However, the paper considered a small dataset and did not specify the data source used. Haque and Abdelgawad [38] also provided binary classification using transfer learning, which is a technique to initialize the weights of a network based on a model pre-trained on a larger dataset and then fine-tuning it according to new and specific information [39]. Although the authors analyzed two distinct sets of images, only one database was used in each class, which may bias the created model. When considering one database for ‘no-finding’ patients and the other for COVID-19 infected ones the model may only distinguish aspects of the two different databases and not intrinsic characteristics of COVID-19 infections from ‘no-finding’ related ones. This potential inconsistency may also be present in other works [40, 41]. These authors evaluated several models also using transfer learning considering one database for COVID-19 and another database for ‘no-finding’ patients with the former work also presenting the problem of highly unbalanced classes. Wang *et al*. [42] is another example of binary classification with few databases, in addition to the limitation of performing one test analysis adopting images already used in the training phase. Hemdan, Shouman and Karar [43] used a single database for both classes, but the extremely limited data quantity reduced the generalization of their results.

**Table 1:** Literature usage of CXR in a single database for each class.

Paper	Number of distinct databases			Model/application characteristics Assumptions/Limitations
	No-finding	COVID-19	Pneumonia	
Sedik *et al*. (2020) [37]	+	+	-	• Focused on DA methods.
				• Small dataset (56 images).
				• Used database not specified.
Haque and Abdelgawad (2020) [38]	1	1	-	• Separate two train/test sets:
				Dataset1: two classes, one dataset in each class
				Dataset2: two classes, one dataset in each class
Minaee *et al*. (2020) [40]	1	1	-	• Use of transfer learning.
				• Evaluation of unbalanced test dataset using accuracy.
Narin, Kaya, and Pamuk (2020) [41]	1	1	-	• Use of transfer learning.
				• Test dataset extremely small (10 images for both classes).
Wang *et al*. (2020) [42]	1	2	-	• Use of transfer learning.
				• Test in images already used on train.
Hemdan, Shouman and Karar (2020) [43]	1	1 (same as ‘no-finding’	-	• Use of transfer learning.
				• Test dataset extremely small (10 images for both classes)
Horry *et al*. (2020) [44]	1	1	1 (same as ‘no-finding’)	• Use of transfer learning.
				• Only binary classification
Abbas, Abdelsamea, and Gaber (2020) [45]	1	1	1 (same as COVID-19)	• Use of transfer learning.
				• Evaluation of unbalanced test dataset using accuracy.
				Use of DA before train/test split.
Khan, Shah and Bhat (2020) [46]	1	1	1 (same as COVID-19)	• Use of transfer learning.
Ozturk *et al*. (2020) [47]	1	1	1 (same as ‘no-finding’)	• Use of transfer learning.
Loey, Smarandache and Khalifa (2020) [48]	2*	1	2* (same as ‘no-finding’	• Use of transfer learning.
				• Do not explicitly define which databases are used in each class.
Apostolopoulos and Mpesiana (2020) [49]	2*	2*	2*	• Use of transfer learning.
				• Absence of test dataset.
				• Only training performance is provided.
Wang, Lin and Wong (2020) [50]	1	4	1** (same as ‘no-finding’)	• Use of a pretrained model on ImageNet images.
Ucar and Korkmaz (2020) [51]	1	1	1 (same as ‘no-finding’)	• Use of transfer learning.
				• Use of DA before train/test split.
This work	4	4	4	• Dedicated CNN (does not require transfer learning).
				• Use of DA after train/test split.
				• Use of Balanced Accuracy (BA) metric for the unbalanced test dataset.
				• Analysis of the effect of image dimension.

* Authors did not clearly mention which databases were used in each class. Therefore, this number is an upper bound.

** More than 99% of this class came from a single database.

+ Not specified

As depicted, most papers presented in the literature use transfer learning methods. For three classes considering ‘no-finding’, pneumonia and COVID-19, Horry *et al*. [44] performed experiments and sensitivity analysis considering the performance of CNN models and transfer learning. However, despite analyzing three classes, only binary classification is presented and only two databases are used in total. In another example with transfer learning, DeTraC network was used with a CNN-based pre-trained model to differentiate the three classes by Abbas, Abdelsamea and Gaber [45]. Despite the interesting use of DA, the authors performed it before separating the used dataset into training and test sets and, therefore, it is expected that an augmented version of an image in both training and testing may inaccurately improve the performance of the model. Khan, Shah and Baht [46], Ozturk *et al*. [47] as well as Loey, Smarandache and Khalifa [48] used transfer learning to classify CXR images in ‘no-finding’, pneumonia and COVID-19. However, as commonly seen, the first two papers considered only two databases in total, with only one database used in COVID-19 class. Loey, Smarandache and Khalifa [48] also used one database for COVID-19 class and despite the possible use of two databases considered for the remaining classes (the paper is not clear about that), the authors adopted these same databases for both classes. Apostolopoulos and Mpesiana [49] used transfer learning for tuning, and evaluated the effectiveness of well-known CNN-based models but also considered three distinct outputs: common bacterial pneumonia, COVID-19, and ‘no-finding’. The authors used several databases to create two datasets on their own; however, once again, each class comes from separate databases.

Wang, Lin and Wong [50] created COVIDx, a public dataset currently generated by combining and modifying five data repositories for COVID-19. The same authors also propose COVID-Net, an open access model, to differentiate ‘no-finding’, non-COVID-19 infection (e.g., viral, bacterial, etc.) from and COVID-19 viral infection. Their model presented better performance than popular VGG-19 and ResNet-50 models. A previous version of COVIDx dataset in which only two specific databases were available was used by Ucar and Korkmaz [51] to create a Bayesian optimization for network parameter estimation and then classify the three classes. Once again, those authors used transfer learning to fine-tune their model and then used only two databases—each one with a specific image type (either ‘no-finding’ or COVID-19 related). In addition, the authors also performed DA before train/test split, which unrealistically improve the performance. In fact, the inadequate use of data, either in the division of databases or in the use of DA, is a problem that may result in misleading diagnostic conclusions, which, may eventually misguide health professional’s decision-making in managing patients.

Therefore, based on the currently available information, there is a lack of AI-based models created on open databases with radiological images from different sources and that do not rely on pre-trained networks (e.g., ResNet, VGG, DeTraC, SqueezeNet). The former approach would provide robustness for the developed data-driven models in detecting specific features of COVID-19 infections rather than just distinct databases. The present work aims to fill these mentioned gaps.

## 3 Methodology

### 3.1 Databases

As previously mentioned, COVIDx is a CXR collection composed of five different repositories: (i) COVID-19 Image Data Collection [52], (ii) Fig 1 COVID-19 Chest X-ray Dataset Initiative [53], (iii) Actualmed COVID-19 Chest X-ray Data Initiative [54], (iv) RSNA Pneumonia Detection Challenge Dataset [55], and (v) COVID-19 Radiography Database [56]. At our data collection step (12-jun-2020), the most recent versions of these five repositories were gathered to form our current dataset of images. Moreover, we added a sixth repository provided in literature: (vi) Large Dataset of Labeled Optical Coherence Tomography (OCT) and Chest X-Ray Images [57]. In fact, although five different repositories comprise COVIDx collection, it is important to note that all healthy (i.e., ‘no-finding’) as well as the resounding majority (>99%) of pneumonia patients come from repository (iv); adopting this collection in its raw form could bias the model. Therefore, we also collected ‘no-finding’ and ‘pneumonia’ data from the other mentioned repositories providing a more diverse and realistic dataset (Table 2). CT images are scarce in public repositories, and thus we firstly considered only CXR as the radiological input image. All repositories were openly available on the internet and maintained by their respective owners or creators. None of the data considered in this work contained personal identifiable information. The authors of this work did not directly contact nor collected data from the patients.

**Table 2:** Data provided by the six different available repositories.

Class	Number of images	Database sources
0—Healthy (‘no-finding’)	10 456	(i), (ii), (iv), (vi)
1—COVID-19	573	(i), (ii), (iii), (v)
2—Pneumonia	11 673	(ii), (iv), (v), (vi)

For our composed database, we gathered 10 451 images of ‘no-finding’ patients, 573 images of COVID-19 patients, and 11 673 images of patients with some type of pneumonia from the six databases mentioned above (Table 2). Instructions to generate the dataset are available and provided as (S1 Dataset).

**Fig 2:**
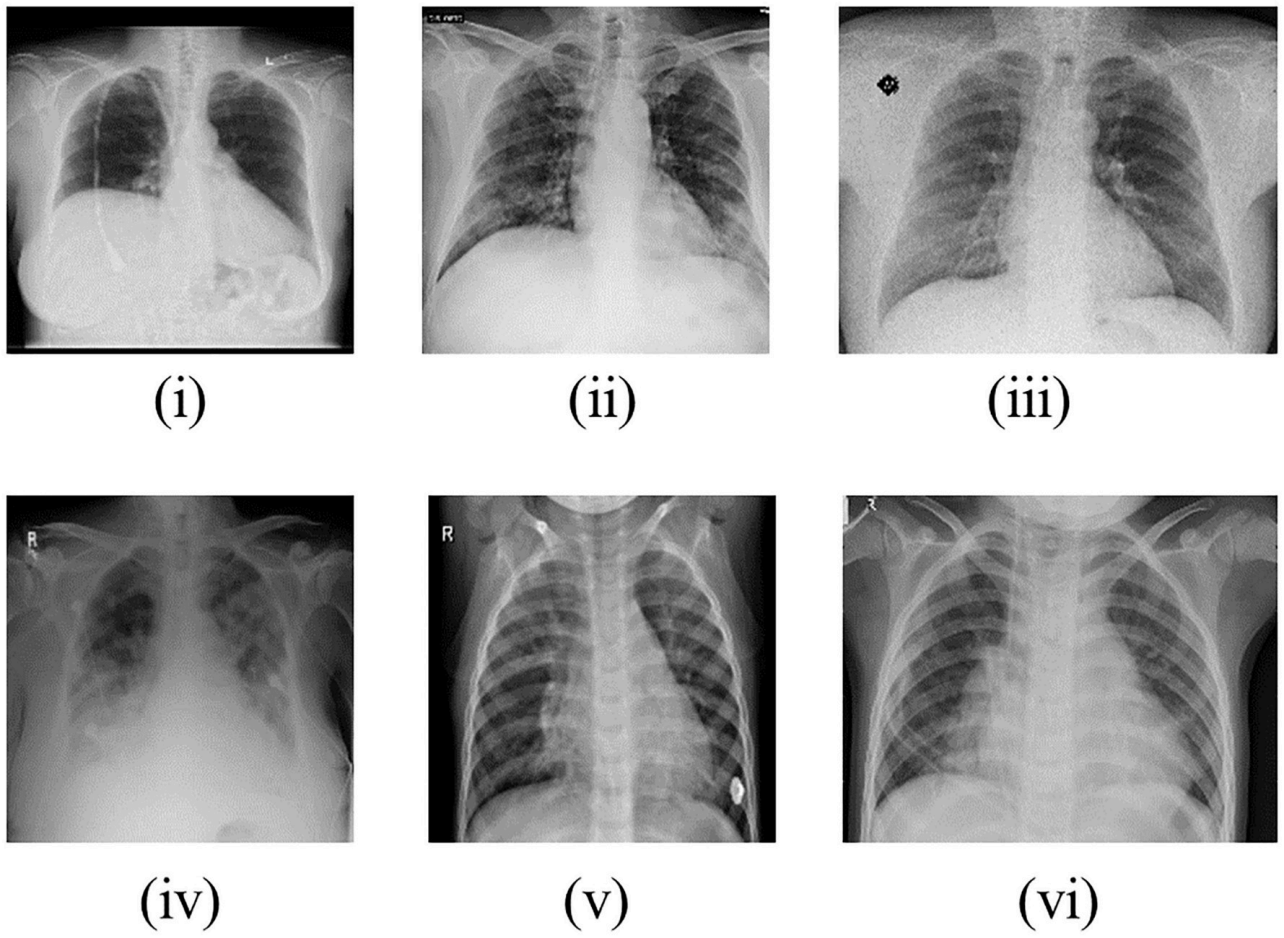
Randomly chosen images from each database used (i-vi).

The lower number of COVID-19-related images is expected because it was an unknown disease until December of 2019. Fig 2 provides a randomly chosen sample of each considered database in which images of repositories (i) and (iv) are from ‘no-finding’ patients; repositories (ii) and (iii) are from patients with COVID-19; and of repositories (v) and (vi) are from patients with pneumonia.

After generating the complete dataset, we split the data in the proportion of approximately 85% for training purposes and the remaining for testing for classes 0, 1 and 2. Here, 10% of the training images were used as a validation set to steer the models’ learning. Due to class 1 (images related to COVID-19 patients), the training dataset is highly unbalanced, and DA is considered here as an alternative to circumvent this issue.

### 3.2 Data Augmentation (DA)

Generating large datasets is either costly, time-consuming, or sometimes simply impossible. It is especially challenging to build big medical image datasets due to the rarity of diseases, patient privacy, the requirement of medical experts for labeling, and the expense and manual effort needed to conduct medical imaging processes [58]. In practice, two techniques are used to grapple with this limitation: transfer learning and DA [39].

As previously mentioned, transfer learning has been the main technique used on the majority of the proposed COVID-19 data-driven diagnostic models to deal with small datasets of CXR. Here, we adopt only the DA approach to avoid dependency on predefined networks and due to the possibility of customizing the augmentation and training of CXR images in a suitable way (e.g., considering distinct input dimensions); this flexibility is not normally present in the pre-trained models of transfer learning.

DA methods artificially increase the size of the dataset producing alternative samples of an image by applying several operations to the original data [59]. When performing DA, it is important to consider the robustness of the method in preserving the same label post-transformation. For example, rotations and flips are generally robust on detection tasks such as cat versus dog, but not robust for digit recognition tasks such as 6 versus 9 [58]. Here, we applied basic operations of DA (e.g., translation, zoom, flips, noise adding) that have been widely used on small datasets for safely combatting overfitting [60] as follows:

- Horizontal flip: flip the images horizontally (i.e., mirror the image);
- Rotation: rotate the images by a random angle between 0.1° and 10°. A minimal zoom is then performed to not require any padding due to rotation;
- Noise injection: add a random value drawn from a statistical distribution to each pixel of the image.

**Fig 3:**
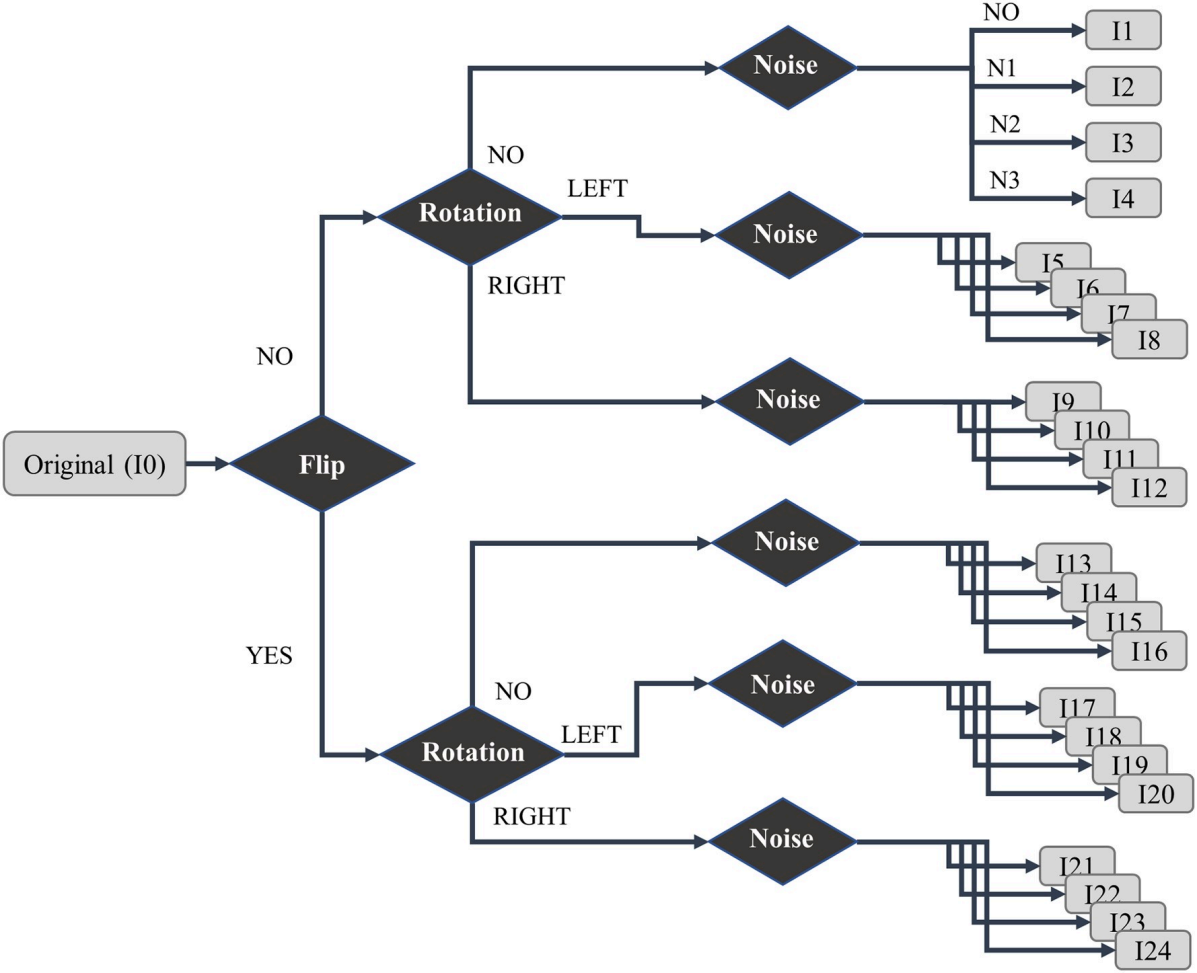
Description of augmented images based on flip, rotation, and noise methods.

Our DA procedure consists of the following steps: (a) flip each original image; (b) rotate both original and flipped images; two new augmented images are generated by rotating to the left and to the right; (c) noises are added in all considered images; three noises were here applied: Gaussian noise (N1), Laplacian noise (N2) and Poisson noise (N3). Indeed, noise can help the CNNs learn more robust features and speed convergence [61]. In summary, with the DA procedure, each original image generates 23 augmented versions (Fig 3).

DA was only applied to COVID-19-related images since this class is the unbalance source of the entire dataset. It is also important to mention that DA is carried out after the separation of the dataset into training and test portions and is only applied to the training CXR images. Therefore, all images in test set are unseen and in their original form (i.e., no DA). Indeed, because DA is only performed on training, the model’s performance (which considers only the test dataset) is not biased to identify augmentation features. Rather, COVID-19 features are presented on both original and augmented images. A total of 33 581 images were considered with 30 544 for training and 3037 for testing. The number of train and test images in each class is presented in Table 3. The steps to generate the complete dataset, including the DA-created images, is available and provided in detail in the supporting information of this paper (S1 Dataset).

**Table 3:** Images available for training and test, after da in covid-19-related training images.

	Train	Test	Total
0– Healthy(‘no-finding’)	9228	1228	10 456
1– COVID-19 (with DA in training images)	11 352	100	11 452
2– Pneumonia	9964	1709	11 673
Total	30 544	3037	33 581

### 3.3 Network architecture

In this section, we describe each layer considered for our proposed networks as well as their respective parameters.

#### 3.3.1 Input layer

The input layer basically depends on the dimension of the images. In our network, all images must have the same dimension presented as a grayscale (single color channel) image.

#### 3.3.2 Batch Normalization layer

Batch normalization converts the distribution of the inputs to a standard normal distribution with mean 0 and variance 1, avoiding the problem of gradient dispersion and accelerating the training process [62].

#### 3.3.3 Convolutional layer

Convolutions are the main building blocks of a CNN. Filter kernels are slid over the image and for each position the dot product of the filter kernel and the part of the image covered by the kernel is taken [63]. All kernels used in this layer are 3 × 3 pixels. The chosen activation function of convolutional layers is the rectified linear unit (ReLU), which is easy to train due to its piecewise linear and sparse characteristics.

#### 3.3.4 Max pooling layer

Max pooling is a sub-sampling procedure that uses the maximum value of a window as the output [64]. The size of such a window was chosen as 2 × 2 pixels.

#### 3.3.5 Fire layer

**Fig 4:**
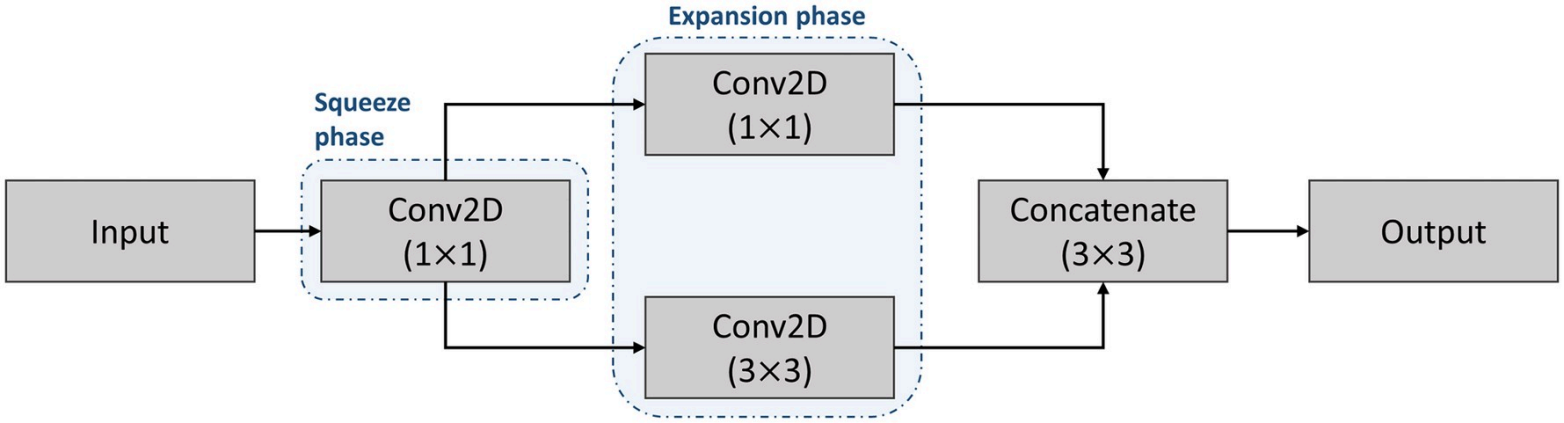
Architecture of the fire layer.

A fire module is comprised of a squeeze convolutional layer (which has only 1 × 1 filters) feeding into an expand layer that has a mix of 1 × 1 and 3 × 3 convolution filters [65]. The use of a fire layer could reduce training time while still extracting data characteristics in comparison with dense layers with the same number of parameters. The layer is represented in Fig 4 in which Input and Output have the same dimensions.

#### 3.3.6 Flatten layer

Flattening reshapes the input into a single-dimension vector that connects the convolutional and dense layers [64].

#### 3.3.7 Dense layer

In a dense (also known as fully connected) layer, all nodes are connected to output nodes of the previous layer. This stems from traditional neural networks or multilayer perceptron (MLP). Thus, a dense layer causes spatial information to be removed. In CNN, dense layers are often used to map spatial features to image labels [63].

#### 3.3.8 Dropout layer

Overfitting is a possible issue due to the enormous number of parameters especially in dense layers. The key idea of dropout is to disregard a part of nodes randomly with probability 1−*p* at each training stage. Therefore, the networks after implementing dropout are different from each other and become thinner than the original neural net. This enhances the model’s resistance to overfitting and makes training faster [62].

**Fig 5:**
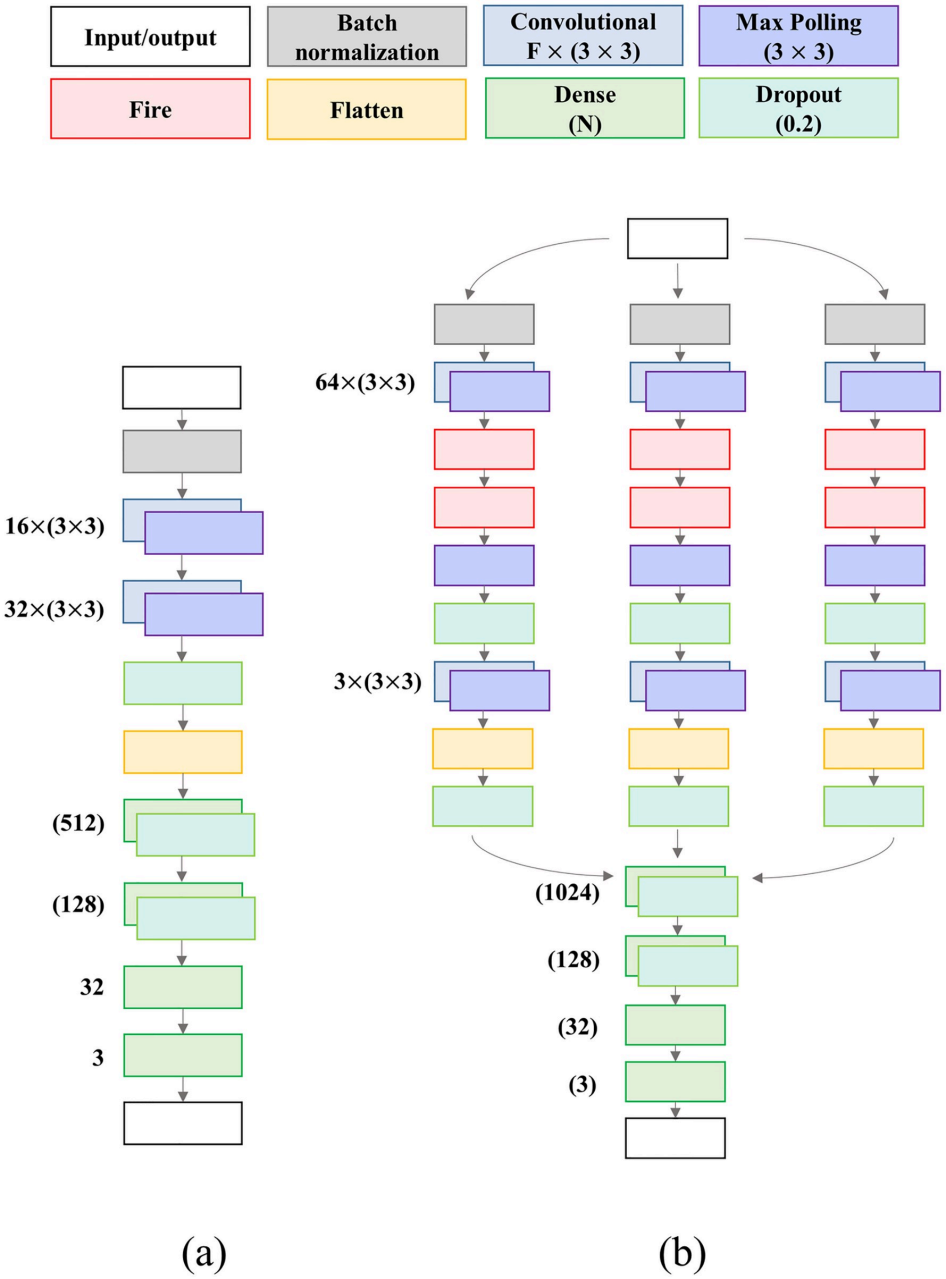
CNN architectures for the two considered models. (a) Model A. (b) Model B.

Two distinct architectures for CNN-based models are considered here:

- Model A: a vanilla model composed in a sequential organization. It starts with a Batch Normalization layer, followed by two Convolutional-Pooling layers and one Dropout layer. Then, flatten is performed and the classification is achieved at the end of three Dense layers (Fig 5a).
- Model B: a more elaborated model composed by three paths. At the beginning, the three branches are separated, following the same architecture in each, starting with a Batch Normalization layer. Then, one Convolutional-Pooling layer is considered, followed by two Fire layers. Then, another Convolutional-Pooling layer is presented, followed by a Flatten and a Dropout layer. Finally. the output of each path is concatenated, followed by a sequence of three Dense layers at the end of the complex structure (Fig 5b).

For each model architecture, we tested several network topologies, and the one which best performed was selected. Note that three symmetrical paths are present in model B. Ideally, each path would update its weights to be related to a specific class. Both architectures have batch normalization and dropout layers. All models were developed using TensorFlow in Python running on a single Nvidia GeForce RTX 2060 Super (8 GB). Most of other layer parameters and training hyperparameters (e.g., kernel size, pool size, learning rate, slope) were determined empirically inspired by well-known values from the literature. The number of epochs for training was 50 with an early stop if the validation loss (i.e., categorical cross-entropy) did not improve in five consecutive epochs. We considered a starting learning rate of 0.001 with linear decay (slope of 0.5) after 10 epochs, and Adam optimizer. The batch target for all models was 32, however, due to computational restrictions, it may be reduced to the maximum 2^*n*^ factor available. This reduced In Fig 5, ‘F’ represents the number of features for a Convolution layer (with kernel size 3 × 3), and ‘N’ represents the number of neurons in a dense layer. All max pooling layers have pool size (3 × 3), and all dropout layers consider a probability of 0.2.

### 3.4 Dimension impact

**Fig 6:**
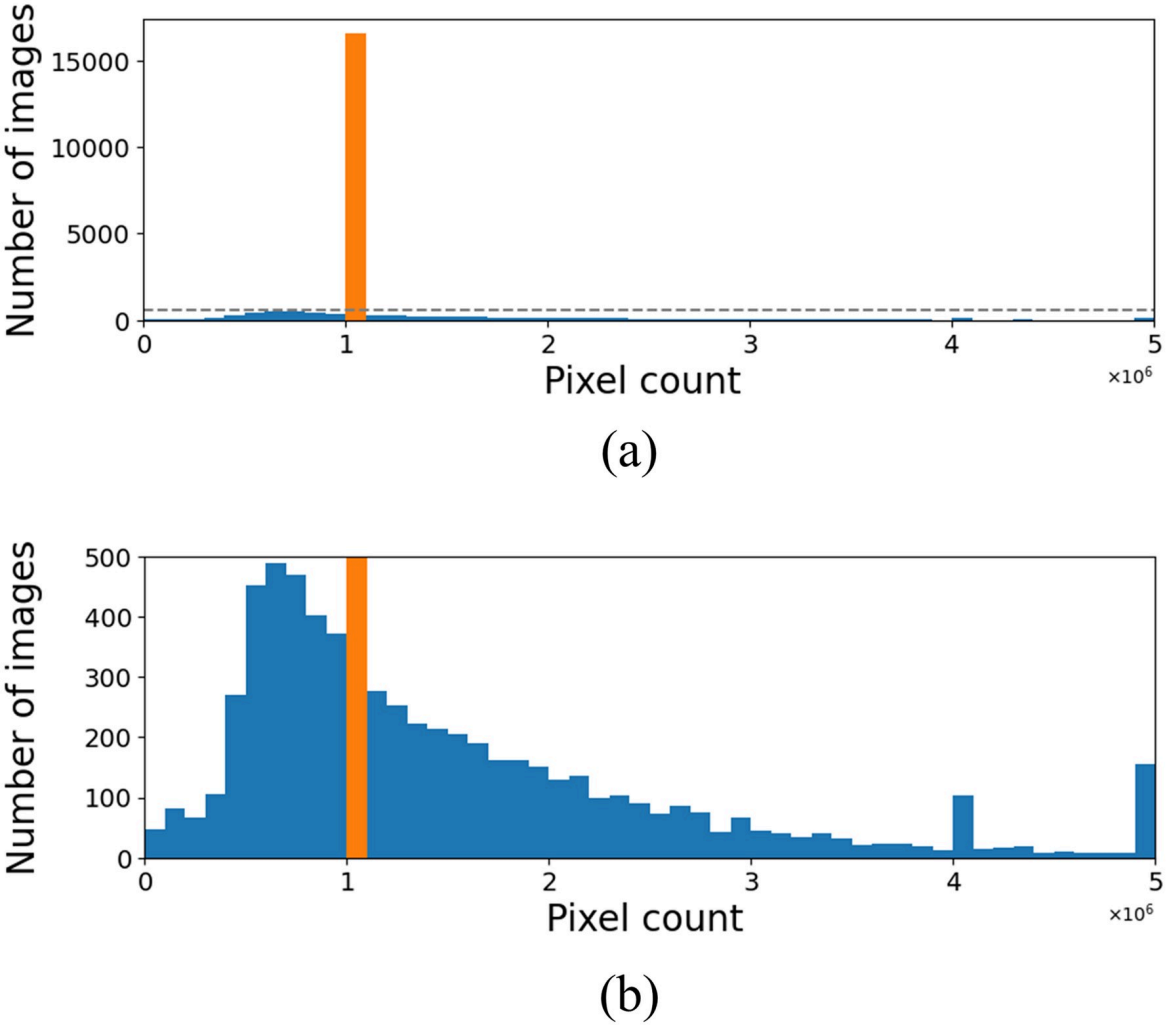
Distribution of the number of pixels in the considered images. Values in 10^6^. (a) Absolute values. (b) Zoomed into the highlighted region.

As previously mentioned, the images described in Table 3 come from six different databases in which an important characteristic is related to their dimensions that vary not only from each database but also within each database. For example, the image dimension goes from 156 × 156 pixels up to 5623 × 4757 pixels. Fig 6 plots histograms of the size (width × height) of all images presented in the dataset. In Fig 6a, it is clear that most images (i.e., 16 259) have a size of 1 048 576 pixels (i.e., 1024 × 1024), but the zoom in Fig 6b shows that there are several other image sizes.

Every input (i.e., image) must have the same dimensions to process CNN models, which requires resizing each image to a default size. However, the dimensions of an image could strongly impact model performance, given that more (less) data is provided to the algorithm in a higher (smaller) size. Thus, we analyzed the models’ performance when the dimensions vary in four different cases: (a) 512 × 512; (b) 768 × 768; (c) 1024 × 1024; and (d) 1536 × 1536. We emphasize this flexibility for the evaluation is only possible because pre-trained models are not used. While this process guarantees that the model always receives the specific image dimensions, it allows for choosing the adequate dimensions before the model is trained.

### 3.5 Evaluation metric

Despite the unbalance in the test dataset, we considered the model accuracy as the average of the correct performance inside each class. This is represented by the balanced accuracy (BA). Represented in Eq (1), BA is a useful metric when the classes in the test set are imbalanced [66] where *P*_*i*_ is the correct number of predictions of class *i* = 0, 1, 2, and *TP*_*i*_ is the total number of images in class *i*. In this case, our three classes are ‘no-finding’, ‘COVID-19’, and ‘pneumonia’.

$$\begin{array}{c} BA=\frac{1}{3}(\frac{P_{0}}{TP_{0}}+\frac{P_{1}}{TP_{1}}+\frac{P_{2}}{TP_{2}}) \end{array}$$ (1)

**Fig 7:**
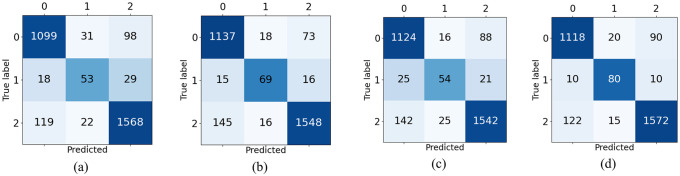
Confusion matrices of Model B when dimension varies. (a) 512 × 512. (b) 768 × 768. (c) 1024 × 1024. (d) 1536 × 1536.

Therefore, even if class ‘COVID-19’ has only 100 images, the performance in this class and in the other two classes (‘no-finding’ and ‘pneumonia’) equally impact the overall model performance. To illustrate, Fig 7b presents the results of one of the evaluated models (i.e. Model B, 768 × 768). Although the number of correctly predicted images in each class is mostly different (i.e., 1137, 69, and 1558), the BA computed is 84.1%, which represents the average of the correct prediction in each class (92.6%, 69.0% and 90.5%). If one directly computes the accuracy, then the performance is 90.9%. Note that the poor performance in Class 2 worsens the BA, as expected.

## 4 Results and discussion

As previously mentioned, the target batch size is 32. However, due to dimension changes, this target is not always achieved. In fact, for Model A with a simple architecture, we used dimensions 512 × 512, 768 × 768, and 1024 × 1024. A batch size of 16 is considered for 1536 × 1536. Model B is more complex and requires more computational resources. In this case, the batches considered were 32, 8, 4, and 2 for dimensions 512 × 512, 768 × 768, 1024 × 1024, and 1536 × 1536, respectively. All comparisons are based on the same test dataset.

### 4.1 Architecture complexity evaluation

Model A and Model B have similar performance in the low/intermediate dimensions (Table 4). In fact, if less information is provided, then there is no need to use more complex architectures that require higher computational effort. However, as the dimension (i.e., available information) rises, Model B outperforms Model A (vanilla model). For the 1536 × 1536 dimension, Model B reaches more than 87.5% of BA; by considering non-COVID classes (i.e., 0 and 2), the correct predictions correspond to more than 91.5%. Also, both methods consistently achieved better performance when dealing with 768 × 768 and 1536 × 1536 rather than 1024 × 1024 dimension. This confirms that image size indeed impacts model performance despite its original dimension.

**Table 4:** BA for Models A and B when image dimension varies.

	Dimension			
	512 × 512	768 × 768	1024 × 1024	1536 × 1536
Model A	79.6%	84.5%	80.9%	83.0%
Model B	78.1%	84.1%	78.6%	87.7%

In Fig 7a–7d, one notices that the worst class performance is always provided in class 1 (‘COVID-19’) predictions despite the dimension. This could be related to the difficulty in detecting subtle nuances of the disease from the considered dataset even with DA. We see that a better overall performance is directly related to better performance in class 1. Fig 7a–7d depicts the performance for model B, but a similar pattern is also seen for Model A.

**Fig 8:**
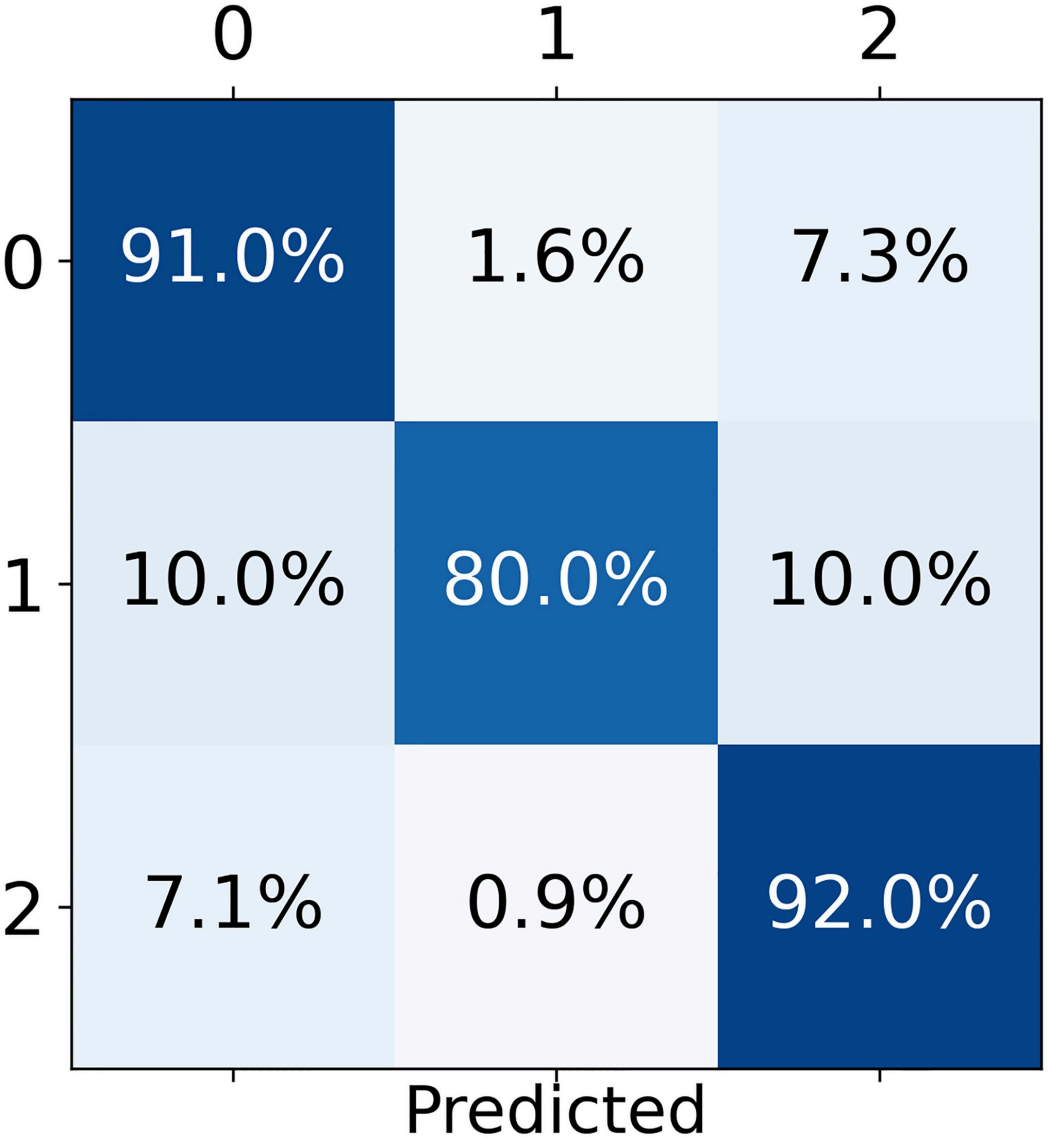
Percentage predictions for the best model. Model B with dimensions 1536 × 1536.

Moreover, an interesting response is seen in Fig 8, considering the best model (i.e., model B—1536 × 1536). The model presents a balanced precision of 97.0% for COVID-19 class given by the proportion of cases for which the model correctly predicted COVID-19 over all COVID-19 predictions (i.e., percentage of true COVID-19 cases over the sum of the second column of Fig 8). Therefore, if the model predicts a COVID-19 infection, then it has almost 97.0% probability of being correct. In fact, this result indicates that the presented model can be used for patient screening, given that a positive prediction triggers a further investigation, clinical diagnosis, and/or treatment.

In fact, for each input, a CNN model produces a probability for each class (i.e., the odds that the considered input belongs to a class), which, in our case, are the probabilities related to ‘no-finding’, ‘COVID-19’, and ‘pneumonia’. The predicted class is the one with the highest probability. Here, we can also analyze specific patterns on these probabilities and not only the class itself.

**Fig 9:**
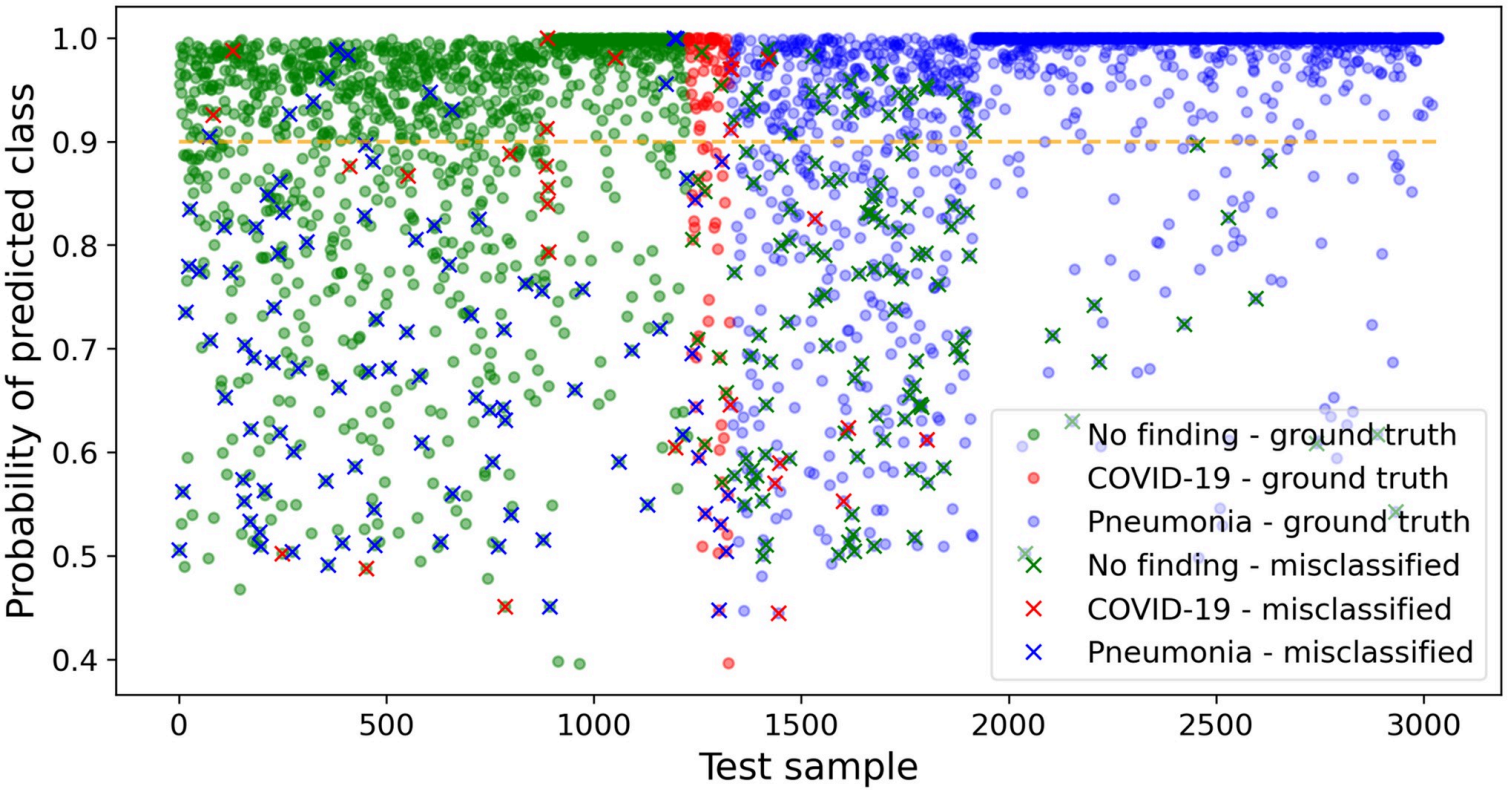
Probabilities for each test prediction. A cross (‘x’) denotes a misclassification.

Fig 9 depicts the probability of each test prediction of our best model (i.e., model B—1536 × 1536). The color of each dot indicates the ground truth class for that sample; a cross over a dot indicates a misclassification with the color of such cross corresponding to the predicted class. For example, a blue cross in the ‘no-finding’ region (i.e., green) means that the model erroneously classified a ‘no-finding’ image as ‘pneumonia’.

**Fig 10:**
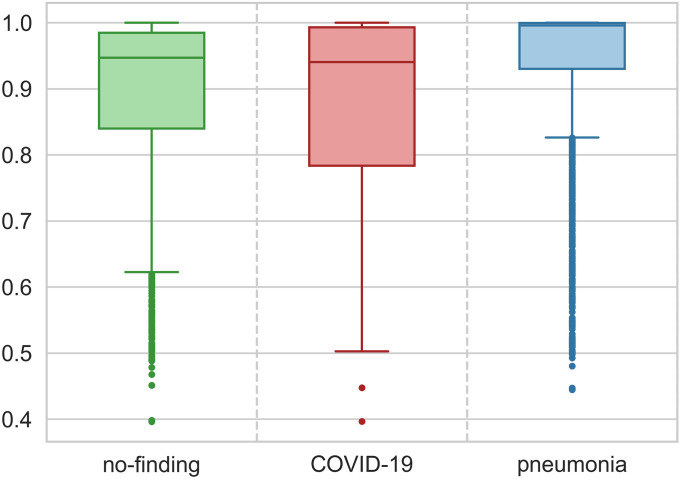
Boxplot for prediction probability of each class.

At first glance, one may infer that most prediction errors are related to classes 0 and 2 because the absolute number of images in each class of test dataset is also unbalanced (see Table 3). However, Fig 10 shows that the probabilities for class 2 (‘pneumonia’) test predictions present the highest median and the smallest variability when compared to the probabilities’ behavior of the other classes. Despite this relatively high precision, there are many (14.5%) ‘outliers’ (i.e., points farther than 1.5 interquartile distance from the first quartile), which increases the uncertainty over the predictions of ‘pneumonia’.

A similar analysis is valid for the ‘no-finding’ class but with a smaller median, greater variability, and fewer outliers (7.0%) than the ‘pneumonia’ counterparts. In fact, the outliers represent an irregular pattern on data, and deeper analysis by a clinical specialist would be recommended for those cases. ‘COVID-19’ probabilities are more disperse with the first quartile ranging from 50% to almost 80% of probability. The high variability of this class may be due to the presence of the subtle details intrinsically related to the novel disease, which might not have been mapped by CNN despite using DA possibly because of the reduced quantity of original ‘COVID-19’ images. Still considering Fig 9, for model output above 90% (i.e., dashed orange line), the accuracy is 96.1% for class 0, 82.8% for class 1, and 99.2% for class 2, which demonstrates great model performance when the output probability related to a given class is high.

### 4.2 Effects of training with fewer databases

**Fig 11:**
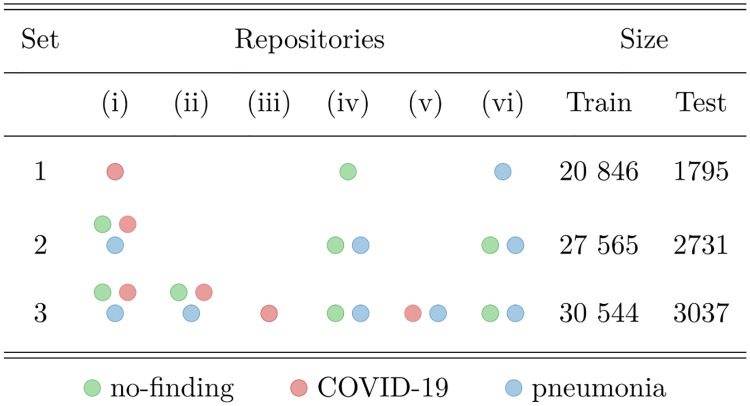
Repository selection for each of the considered sets.

To better understand and compare the effects of not considering multiple dataset sources (repositories), we evaluated several cases of training and testing on specific repositories combinations (see Fig 11 and Table 5).

**Table 5:** Considered cases and respective datasets.

	Train	Test
Case 1	Set 1	Set 1
Case 2	Set 1	Set 3
Case 3	Set 2	Set 2
Case 4	Set 2	Set 3
Proposed	Set 3	Set 3

In Case 1, we trained a model using images from a single repository (Set 1) for each class. For the ‘no-finding’ class, repository (iv) was used; for the ‘COVID-19’ class, repository (i) was considered; for the ‘pneumonia’ class, repository (vi) was selected. As previously discussed, using a single repository for each class may cause the model to be biased towards detecting dataset-specific features, instead of learning the characteristic features of the classes. Table 6 shows the resulting BA for each model and image dimension. Note that these values are higher than when training with all repositories, which could be a sign of learning database features. To further investigate this, in Case 2, we evaluate the BA for these models using Set 3 for testing, which is also displayed in Table 5. As suspected, BA for the complete test set is drastically reduced, indicating bias towards database features (e.g., for dimension 1536 × 1536 BA dropped from 97.2% to 75.7%). Also notice that the relation between dimension and model performance is similar to what is observed in our proposed model results, where performance increases with dimension, but is worse for 1024 × 1024.

**Table 6:** BA for Models A and B in all cases analyzed.

		Dimension			
		512 × 512	768 × 768	1024 × 1024	1536 × 1536
Case 1	Model A	89.1%	91.9%	88.4%	91.1%
	Model B	90.0%	90.6%	84.8%	97.2%
Case 2	Model A	67.9%	71.2%	68.1%	70.2%
	Model B	69.5%	70.3%	67.8%	75.7%
Case 3	Model A	78.0%	78.6%	78.6%	82.7%
	Model B	77.6%	77.3%	83.6%	82.5%
Case 4	Model A	77.4%	79.2%	83.6%	84.4%
	Model B	76.7%	76.7%	79.3%	82.4%
Proposed	Model A	79.6%	85.5%	80.9%	83.0%
	Model B	78.1%	84.1%	78.6%	87.7%

In Cases 3 and 4, the same repositories used in Cases 1 and 2 were used for training, but all classes available in each of these repositories were considered (Set 2). For testing, Case 3 uses Set 2, and Case 4 considers Set 3. Comparing Case 1 with Case 3 (or with Case 4), although more images were present in Set 2 than in Set 1, the model trained and tested on Set 1 resulted in much higher BA, once more suggesting an inflated result, which is a consequence of detecting database-specific features, instead of only CXR characteristics (see Table 6). Comparing Case 2 with Case 4 (i.e., same test set), the latter has greater performance indicating that mixing repositories in training is indeed beneficial. Cases 3 and 4 had similar performance which is explained by the fact that mixing the three repositories used in Set 2 (i.e., (i), (iv) and (vi)) covers the most representative characteristics for the three classes. Indeed, Set 3 is only 10.8% larger than Set 2, in number of training images.

Finally, we evaluated our best model (1536 × 1536, Model B, trained on Set 3) in other two cases, using test images from Set 1 (Case 5) and Set 2 (Case 6). For Case 5, the model achieved a BA of 88.9%, indeed avoiding overfitting due to detecting databases. For Case 6, the BA of 86.6% is comparable to the performance in Set 3 (87.7%), once again explained by the fact that Set 2 contains most images from the complete dataset (Set 3).

### 4.3 Binary classification

Some interesting results emerge if we consider a binary classification. For example, Table 7 displays class ‘no-finding’ and class ‘finding’ for the detection of any type of disease (i.e., COVID-19 or pneumonia). Note that with this classification, the precision, accuracy, false omission rate, specificity, sensitivity, geometric mean (GM), and Youden’s Index (YI) are 91.0%, 91.1%, 8.6%, 91.0%, 91.0%, 91.0%, and 82.0% respectively. These are important results to identify a patient that will require treatment.

**Table 7:** Confusion matrix, in %, considering ‘no-finding’ vs. ‘finding’ binary classification.

		Prediction	
		‘no-finding’	finding
Ground truth	‘no-finding’	91	9
	finding	8.55	91.45

Analogously, the results for binary classification to exclusively detect COVID-19 is seen in Table 8 (‘not COVID-19’ vs. COVID-19). Our best model achieved a precision, accuracy, false omission rate, specificity, sensitivity, GM, and YI equal to 98.4%, 89.4%, 16.8%, 98.7%, 98.5%, 98.6%, and 97.2% respectively. Indeed, as previously mentioned, the model has strong performance when predicting a COVID-19 infection (represented by the precision).

**Table 8:** Confusion matrix, in %, considering ‘not covid’ vs. ‘covid’ binary classification.

		Prediction	
		not COVID	COVID
Ground truth	not COVID	98.75	1.25
	COVID	20	80

### 4.4 Comparison of results

#### 4.4.1 DA impact

**Fig 12:**
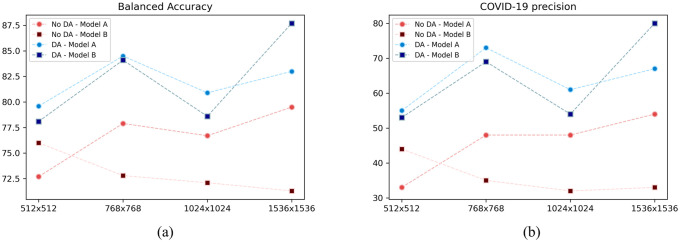
Comparison of Models A and B with and without DA. (a) Balanced accuracy. (b) COVID-19 precision.

In our model, DA is performed only for COVID-19 class. However, the entire training process (and test performance) changes when new evidence (i.e., data) is aggregated despite being related to just one class. Note from Fig 12 that the overall performance in all considered models and dimensions are worse without DA. However, we can generally see that the most discrepant performance is indeed in COVID-19 class, which is the actually augmented class. Moreover, without DA, Model A and Model B have similar performance in all dimension, which is probably related to the fact that Model A could still handle the high-dimension/less-data information.

#### 4.4.2 Comparison and discussion of literature

Finally, we compared our model with other proposed in the literature. Although we previously argued that accuracy is not a suitable metric for unbalanced datasets, which is common in COVID-19 research, we present it here for all models described in Table 1 because often this is the presented performance metric and we cannot calculated more suitable ones (e.g., BA).

Firstly, one has to perform a critical analysis on results presented in Table 9. As expected, the models have higher performance in binary classification than in 3-class classification. However, as depicted in Table 1, note that in the binary classification, often one database is considered for each class, which naturally inflates results. We presented in Section 4.2 our inflated model trained with one repository of each class, achieving a BA of 97.2%. Here, we calculate the accuracy of this model for binary and 3-class prediction. The model has 98.33% for 3 classes and 98.56% for 2 classes. These results are comparable to all results presented in Table 9, which are also performed in single database.

**Table 9:** Accuracy of models proposed by the literature.

Paper	2 classes	3 classes
Sedik *et al*. (2020) [37]	99.00%	-
Haque and Abdelgawad (2020) [38]	98.30%	-
Minaee *et al*. (2020) [40]	92.29%	-
Narin, Kaya, and Pamuk (2020) [41]	98.00%	-
Wang *et al*. (2020) [42]	99.33%	-
Hemdan, Shouman and Karar (2020) [43]	90.00%	-
Horry *et al*. (2020) [44]	87.87%	-
Abbas, Abdelsamea, and Gaber (2020) [45]	-	95.12%
Khan, Shah and Bhat (2020) [46]	99.00%	89.60%
Ozturk *et al*. (2020) [47]	98.08%	87.02%
Loey, Smarandache and Khalifa (2020) [48]	100.00%	81.48%
Apostolopoulos and Mpesiana (2020) [49]	96.78%	94.72%
Wang, Lin and Wong (2020) [50]	98.30%	95.00%
Ucar and Korkmaz (2020) [51]	99.78%	98.30%
Our inflated model	98.56%	98.33%
Our proposed model	98.19%	91.21%

In addition, considering models with similar performance for 2- and 3-class classification, papers commonly have some tricky characterization. For example, Apostolopoulos and Mpesiana (2020) [49] presented only the training accuracy. Despite being used by other authors for comparison, training performace should not be used as benchmark. Ucar and Korkmaz (2020) [51] performed DA before the train/test split; therefore, an augmented version of an original image is certainly presented in test. Hence, it is probably easier for the model to correctly classify this augmented image once it knows the true label of the original version. Yet, note that for the other models with 2- and 3-class classification, the performance is strongly reduced from the former to the latter, but not for our inflated model. This is explained because the models used only two repositories for both 2- and 3-class classification. Therefore, in the binary classification, each class is related to only one repository. But for 3-class classification, one repository is the source of two classes, and the model cannot properly differentiate between these two classes within the same repository. Because we created our inflated model with one repository for each class, it still disguises the results for 3 classes, even though we previously explained that, in fact, the model learned repository characteristics. However, our proposed model still presents robust results, created from many repositories and, therefore, representing a realistic and suitable environment.

## 5 Limitations of the study

The proposed model is intended as a support tool. Thus, a detailed clinical study involving pieces of evidence of different sources is necessary to provide a final medical diagnosis.

Additionally, despite the use of DA to artificially generate more than 10 000 images, the number of original images related to COVID-19 cases is rather small (i.e., 573). More images would be desirable and would provide more visual information about COVID-19 features.

Note that evaluating images is not as intensive as training. The assessment of one single image takes less than a second, even with a CPU. However, training the proposed models is a computer-intensive task, which took about 5 to 6 hours to train using a GPU for high image dimensions (Section 3.3). Therefore, training new models based on the proposed methodology may not be feasible in low-end computers.

## 6 Conclusions

The rapid and devastating outbreak of COVID-19 prompted a global challenge for science to develop new diagnostic tests, medicines, and vaccines to tackle this public health problem. This paper describes an alternative data-driven diagnostic method for COVID-19 to support clinical decision-making processes from images and based on CNN models. From the literature review, we identified a limitation on methods that consider few databases to train a CNN model, which may incorrectly inflate the model results.

Therefore, we gathered six databases with CXR images from patients infected with COVID-19 or pneumonia as well as healthy (‘no-finding’). The public dataset of CXR for COVID-19 patients is rather small, and we considered a DA procedure to increase artificially the number of images related to this class. The performance of the CNN-based models has also been impacted by the input dimensions given that images with higher sizes better represent subtle aspects possibly unseen in smaller images. The models were carefully developed to be accurate, simple, robust, and compatible with images from different sources.

Although using single databases we achieve an inflated result of 97.2% for BA, our best, properly trained model had a BA of 87.7% for correctly classifying a CXR image as associated with a ‘no-finding’ or an infected patient differentiating the latter as ‘COVID-19’ or ‘pneumonia’. Moreover, the balanced precision for COVID-19 detection was 97%, which could support clinical diagnosis. After training, the models require less than a second to evaluate an image. Therefore, the model could be used as a first viewpoint to screen and prioritize patients in fully occupied hospitals, especially those with long waiting lines for evaluation. Also, the proposed model can be retrained as more images from COVID-19-positive patients become available. Even better results are expected with such data.

Moreover, the best model provided in this work could be an alternative method for increasing the number of tested individuals for COVID-19. This approach also has value in teleradiology [67]. Indeed, a data-driven diagnostic method integrated into a telehealth system would rapidly classify radiological images, which is especially useful with communicable diseases. Such an approach could filter patients. Only images with no clear class distinction would require in-depth analysis by the imaging expert thus saving time during the decision-making process.

Currently, we have been working on CT images available on the literature. To that end, we have to adjust the network architecture, as well as its parameters, to better suit this data. In addition, due to the scarcity of CT images, a more robust DA method, based on generative adversarial networks, is being developed.

## Supporting information

S1 Dataset Dataset information and instructions for dataset creation. (PDF)
