# Supplementary material for: Convolutional neural network model based on radiological images to support COVID-19 diagnosis: Evaluating database biases
Source: PLoS One. 2021 Mar 1;16(3):e0247839. doi: 10.1371/journal.pone.0247839 (PMC7920391; doi:10.1371/journal.pone.0247839)
Supplement: S1 Dataset — (PDF) [file pone.0247839.s001.pdf]

Dataset information

Creation date: jun-12-2020

Dataset versions for our research

Below, the dataset versions we used in our paper are listed, which were the most up-to-date available versions at the time of dataset creation. We provide the source links and, when possible, specific links to the used versions.

- covid-chestxray-dataset
    - GitHub repository
    - Commit hash: 9b9c2d5f175107d211d1a62ce9b317509b50f855
    - Last change: jun-12-2020
- Figure1
    - GitHub repository
    - Commit hash: 95bb569e55f4110d3b734c0f355340e04e16989a
    - Last change: may-08-2020
- Actualmed:
    - GitHub repository
    - Commit hash: 27240dd8dec9454bc97f9f3c0c432ef434de2517
    - Last change: may-05-2020
- RSNA Pneumonia Detection Challenge
    - Kaggle dataset - Stage 2 update.
    - Download date: may-14-2020
- COVID-19 Radiography Database
    - Kaggle dataset - Version 1
    - Download date: may-14-2020
- Large Dataset of Labeled Optical Coherence Tomography (OCT) and Chest X-Ray Images
    - Mendeley dataset
    - Version 3. DOI: 10.17632/rscbjbr9sj.3
    - published: jun-01-2018

Steps to create an up-to-date version of the dataset

For creating an up-to-date version of such dataset, one can follow the steps below.

1. Download source data.
    - covid-chestxray-dataset
    - Figure1
    - Actualmed
    - RSNA Pneumonia Detection Challenge
    - COVID-19 Radiography Database
    - Large Dataset of Labeled Optical Coherence Tomography (OCT) and Chest X-Ray Images
2. Select and add images to the new dataset.
    - No finding

    - covid-chestxray-dataset
    - Figure1
    - RSNA Pneumonia Detection Challenge
    - Large Dataset of Labeled Optical Coherence Tomography (OCT) and Chest X-Ray Images
  - COVID-19
    - covid-chestxray-dataset
    - Figure1
    - Actualmed
    - COVID-19 Radiography Database
  - Pneumonia
    - Figure1
    - RSNA Pneumonia Detection Challenge
    - COVID-19 Radiography Database
    - Large Dataset of Labeled Optical Coherence Tomography (OCT) and Chest X-Ray Images
3. Resize all images to desired dimensions.
  - 512x512
  - 768x768
  - 1024x1024
  - 1536x1536
4. Perform data augmentation.
  - Flip
  - Rotate left/right
  - Add noise
    - Gaussian noise
    - Laplacian noise
    - Poisson noise

Datasets used for comparison

In order to create the datasets we used for comparison in the paper, the instructions below can be followed.

- Dataset without data augmentation: follow the steps above, excluding step 4.
- COVIDx: Instructions for creating COVIDx v3.
